# Dietary Behavior and Risk of Depression: Effects of Ultra-Processed Food and Water Intake in a National Sample of the United States

**DOI:** 10.14740/jocmr6448

**Published:** 2026-02-28

**Authors:** Rahul Menon, Krystal Hunter, Satyajeet Roy

**Affiliations:** a Cooper Medical School of Rowan University, Camden, NJ, USA; b Department of Medicine, Cooper University Health Care, Camden, NJ, USA

**Keywords:** Depression, Ultra-processed food, Hydration, Water intake and diet, Mental health disparities

## Abstract

**Background:**

Diet is increasingly recognized as a modifiable determinant of mental health. High intake of ultra-processed foods (UPFs) can be associated with poor psychological outcomes; however, the protective role of hydration, particularly plain water intake, remains underexplored. We aimed to evaluate the independent and combined associations of UPF and water intake with moderate-to-severe depression among the adult population of the United States (US).

**Methods:**

We analyzed cross-sectional data from the National Health and Nutrition Examination Survey (NHANES) 2021–2023. UPF intake was proxied using the percentage of daily calories from added sugars and categorized into quartiles. Water intake (g/day) was similarly categorized into quartiles. Moderate-to-severe depression was defined as a Patient Health Questionnaire-9 (PHQ-9) score ≥ 10. Survey-weighted logistic regression models assessed associations between diet exposures and depression risk, adjusting for age, sex, and body mass index (BMI). Subgroup, sensitivity, and interaction analyses were conducted.

**Results:**

Prevalence of PHQ-9–based depression in our sample was 10.9%. Participants in the highest UPF quartile had higher odds of PHQ-9–based depression when compared to the lowest (odds ratio (OR) = 1.547, 95% confidence interval (CI): 1.545–1.550, P < 0.001). Conversely, those in the highest water intake quartile had lower odds of PHQ-9–based depression (OR = 0.486, 95% CI: 0.486–0.487, P < 0.001). The UPF–water interaction was statistically significant but of minimal clinical relevance. Subgroup analyses showed more severe vulnerabilities to depression from UPF consumption among males, Black and Hispanic individuals, and those with lower educational attainment. A small but statistically significant interaction (β = –0.07, P = 0.017) indicated that water intake modestly attenuated the UPF–depression relationship. Associations persisted after exclusion of extreme BMI values.

**Conclusions:**

Increased UPF intake is associated with higher risk of depression in the US adults while increased water intake confers a protective effect. These findings underscore the need for dietary strategies that simultaneously reduce UPF intake and promote hydration, with tailored interventions for the high-risk groups.

## Introduction

Depression is a leading cause of global disability, affecting around 350 million people worldwide and contributing to substantial morbidity, impaired quality of life, and increased mortality risk [1]. While pharmacological and psychotherapeutic treatments remain central to clinical management, evidence from systematic reviews and meta-analyses indicates that modifiable lifestyle factors, such as diet quality, may play a significant role in both the onset and progression of depressive symptoms. While experts have expressed a need for more robust evidence, higher adherence to healthy dietary patterns, such as the Mediterranean diet, could be associated with a lower risk of developing depression, whereas poor diet quality has been linked with increased risk of depression [2–4].

Ultra-processed foods (UPFs), characterized by the Nova classification system as industrial formulations rich in added sugars, fats, sodium, and synthetic additives, are now pervasive in the diet in the United States (US) [5]. These foods typically include sugar-sweetened beverages, packaged snacks, reconstituted meat products, and ready-to-eat meals. Multiple large-scale epidemiological studies have linked higher UPF intake with increased risk of depression [6, 7]. Although causal pathways remain under investigation, proposed mechanisms include systemic inflammation, gut microbiota disruption, oxidative stress, and insulin resistance [8–11]. It has been reported that UPFs account for 57.9% of total energy intake and contribute 89.7% of the energy intake from added sugars in the US diet, during the National Health and Nutrition Examination Survey (NHANES) survey period from 2009 to 2010 [12]. A study reported that in the US children aged 2 to 19 years, UPFs contributed 65% of total energy intake and 92% of energy from added sugars from the survey period of 2009 to 2014 [13]. Given that UPFs likely account for a high percentage of added sugar intake in the US diet, the percentage of total calories from added sugars could act as a reasonable proxy for UPF consumption in population-level dietary assessments. Therefore, in this study, we used percent of total calories from added sugars as a practical proxy for UPF consumption. While not all UPFs contain added sugars (e.g., processed meats, savory snacks), the strong overlap between added sugar consumption and UPF intake supports the use of added sugars as a reasonable proxy when direct Nova-based classification is not implemented.

Although early research works on hydration and mental health were often constrained by small sample sizes, inadequate adjustment for confounders, or limited generalizability, more recent studies, including some large-scale cohort studies and meta-analyses have addressed many of these limitations [14–16]. However, a few studies have simultaneously explored potential protective dietary factors such as plain water intake alongside UPF consumption. Adequate hydration is essential for neurocognitive functioning. Even mild dehydration has been associated with impairments in mood, alertness, and cognitive performance [14–16]. Experimental studies suggest that increased water consumption might improve mood and reduce fatigue and confusion, while a reduced intake could negatively affect calmness, positive emotions, and vigor, highlighting hydration as a potential modulator of emotional well-being [17–19]. However, the role of water intake as an independent predictor of depression, and as a potential modifier of the UPF-depression relationship, remains underexplored in nationally representative samples. Till date, no single study has simultaneously examined the association between UPF consumption, water intake, and depression, particularly within a representative sample of the US adults.

In this study, we aimed to evaluate the independent and joint associations of UPF intake and plain water consumption with moderate-to-severe depression in a nationally representative sample of the US adults using data from the NHANES 2021–2023. By utilizing the percentage of total calories coming from added sugar as a proxy for UPF consumption, we hypothesized that higher UPF intake would be positively associated with depression, while higher water intake would have an inverse association. We also examined whether water intake modified the relationship between UPF consumption and depression, and whether such associations differed across demographic subgroups.

## Materials and Methods

This study was a cross-sectional study in which we utilized the publicly available data from the NHANES 2021–2023. NHANES is a nationally representative survey of the civilian, non-institutionalized US population and it uses a complex, multistage probability design. This study met international ethical guidelines set for human and animal study and was performed in accordance with the Declaration of Helsinki. The dataset included detailed information gathered through in-person health interviews, dietary assessments, and physical examinations conducted by trained personnel. Participants with endocrine disorders, including rare conditions such as diabetes insipidus, were not specifically excluded unless missing exposure, outcome, or covariate data precluded analysis. Participants aged 18 years and older were included in the study if they had complete data for dietary sugar and total caloric intake, total plain water intake, Patient Health Questionnaire-9 (PHQ-9) scores for the assessment of depression [20], in addition to their age, sex, and body mass index (BMI). In a sensitivity analysis, participants with extreme BMI values (defined as less than 18.5 kg/m^2^, or greater than 40 kg/m^2^) were excluded to test the robustness of the observed associations.

UPF intake was operationalized as the percentage of total daily caloric intake derived from added sugars, thereby standardizing exposure relative to total energy intake. Because the exposure was expressed as a proportion of total energy, additional adjustment for total caloric intake was not performed. Further adjustment for macronutrients such as carbohydrate, sugar, or fat was avoided to prevent overadjustment and collinearity, as these components are intrinsically linked to the UPF proxy definition. This continuous variable was divided into low to high quartiles (Q1–Q4) to assess the potential for a dose-response relationship. Water intake was measured as daily plain water consumption in grams, based on dietary recall interviews, and was similarly categorized into low to high quartiles (Q1–Q4). The primary outcome was moderate-to-severe depression, defined as a PHQ-9 score of 10 or greater based on the observation that a PHQ-9 score of ≥ 10 has a sensitivity of 88% and a specificity of 88% for major depression [20]. This was analyzed as a binary variable (yes or no). Covariates included in the adjusted models were age, sex, and BMI. Subgroup and stratified analyses also accounted for self-reported race and ethnicity (categorized as non-Hispanic White, non-Hispanic Black, Hispanic, and other) and education level (less than high school, some college, or college graduate and above).

To address potential loss of information from categorization of continuous predictors, supplementary survey-linear regression models were constructed with log-transformed PHQ-9 scores as the continuous outcome and percent of calories from added sugars (UPF proxy) and total plain water intake (g/day) as continuous predictors. Additionally, an interaction term between UPF and water intake was included to test for effect modification. Sensitivity analyses excluding participants with BMI < 18.5 kg/m^2^ or > 40 kg/m^2^ were repeated using these continuous-variable models.

All statistical analyses were conducted using IBM Statistical Product and Service Solutions (SPSS) Statistics, version 29.0. Descriptive statistics were used to summarize sample characteristics, and median and interquartile ranges (IQRs) were calculated for the variables of UPF intake, water intake, and PHQ-9 scores. Weighted proportions were calculated for categorical variables including quartile distributions and depression prevalence, in accordance with NHANES survey design.

**Figure 1:**
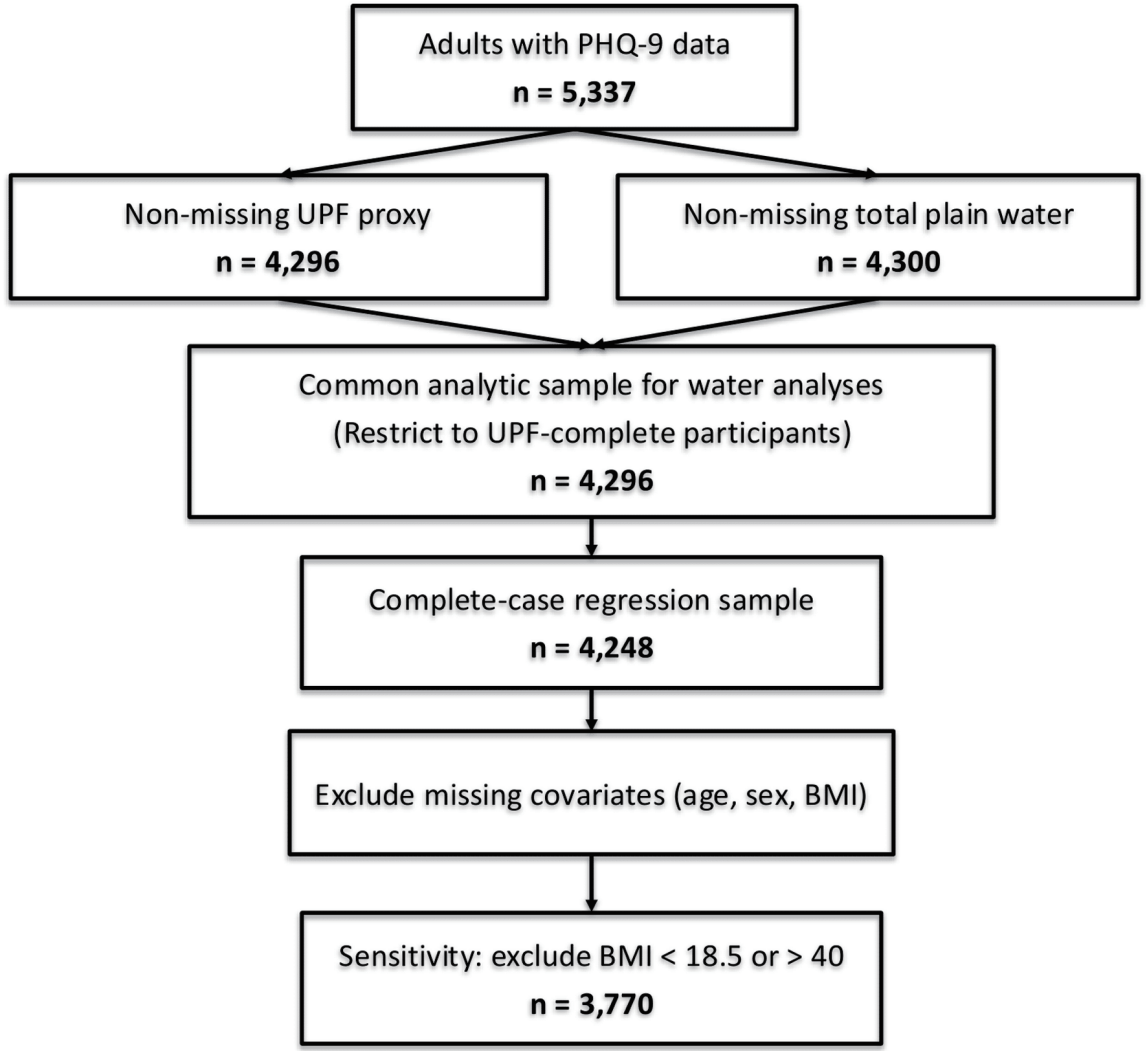
STROBE flow diagram (NHANES 2021–2023).

We followed Strengthening the Reporting of Observational studies in Epidemiology (STROBE) guidance for cross-sectional studies. We report unweighted sample counts at each analytic step and survey-weighted estimates with 95% confidence intervals (CIs). A participant flow diagram details inclusion/exclusion criteria and reasons (Fig. 1). The primary analysis involved survey-weighted logistic regression to evaluate the association between UPF quartile and the presence of PHQ-9–based depression (model 1). A second model included both UPF and water intake quartiles as predictors of PHQ-9–based depression while adjusting for age, sex, and BMI (model 2). Odds ratios (ORs), 95% CIs, and P-values were reported for all associations. To assess the influence of extreme BMI values, sensitivity analyses were conducted by excluding participants with BMI below 18.5 or above 40 kg/m^2^ and rerunning the regression models. Interaction analysis was also performed by including a cross-product term between UPF and water intake quartiles in the regression model to assess whether water intake modified the relationship between UPF and PHQ-9–based depression. The interaction term was interpreted based on statistical significance and comparisons of predicted odds across combinations of exposure levels.

Subgroup analyses were performed by stratifying the sample according to sex, race and ethnicity, and education level. Within each stratum, logistic regression models were used to assess the association between UPF quartile and PHQ-9–based depression, adjusting for age and BMI. To evaluate the independent effect of water intake on PHQ-9–based depression, parallel stratified models were constructed, adjusting for UPF quartile, age, and BMI. As the study used publicly available de-identified data, it was exempt from the Institutional Review Board oversight.

## Results

Of 5,337 adults with PHQ-9 data, 4,296 had non-missing UPF proxy and 4,300 had non-missing total plain water intake. To maintain a common analytic sample, water analyses were restricted to the 4,296 participants with non-missing UPF proxy; after excluding participants missing covariates (age, sex, BMI), the complete-case sample for regression models was 4,248 (Table 1). A sensitivity analysis excluding BMI < 18.5 or > 40 included 3,770 participants. The final weighted analytic sample included 165,710,643 participants (Table 1). The unweighted mean age of all participants in this nationally representative sample was 50.8 (95% CI: 50.2–51.5) years, with a mean BMI of 30.1 (95% CI: 29.8–30.5) kg/m^2^. Males represented 43.8% of this sample. Among the social variables, 14.1% had a history of cigarette smoking, while 18.5% had a history of drinking alcohol. The unweighted prevalence of PHQ-9–based depression was 10.9% (95% CI: 10.0–11.8%) and the unweighted mean PHQ-9 depression score was 3.61 (95% CI: 3.50–3.73). UPF intake, measured as the percentage of daily calories from added sugar, had an unweighted mean of 4.81% (95% CI: 4.74–4.88). Unweighted mean water intake was 2,743 g/day (95% CI: 2,652–2,835) (Table 1).

**Table 1:** Baseline Characteristics of All Participants

Variable	Participants (n = 4,248)
Age (years), mean (CI)	50.8 (50.2–51.5)
Cigarette smoking, % (CI)	14.1 (11.9–16.3)
Alcohol drinking, % (CI)	18.5 (16.6–20.3)
Gender (males), % (CI)	43.8 (42.4–45.4)
BMI (kg/m^2^), mean (CI)	30.1 (29.8–30.5)
PHQ-9 score, mean (CI)	3.61 (3.50–3.73)
PHQ-9–based depression prevalence, % (CI)	10.9 (10.0–11.8)
UPF intake: % daily calories from added sugar, mean (CI)	4.81 (4.74–4.88)
Water intake (g/day), mean (CI)	2,743 (2,652–2,835)

BMI: body mass index; CI: confidence interval; n: number of subjects; PHQ-9: Patient Health Questionnaire-9; UPF: ultra-processed food.

The weighted NHANES data, which adjust the unweighted data to represent a national sample, were analyzed to find the frequencies for each quartile of UPF and water intake (Table 2). The highest weighted frequency of participants in the UPF quartiles was seen in quartile 1 that represented the lowest UPF intake (27.9%), which decreased progressively to quartile 4 that represented highest UPF intake (23.1%). Conversely, weighted frequency for water intake was highest in quartile 4 that represented highest water intake (30.6%) and lowest in quartile 1 that represented lowest water intake (20.7%) (Table 2). While the prevalence of PHQ-9–based depression increased from the lowest to the highest quartile of UPF intake and decreased from the lowest to the highest quartile of water intake, there were no clear dose-response relationship between the prevalence of PHQ-9–based depression and the quartiles of either UPF intake or water intake (Table 3).

**Table 2:** Quartile Based Frequencies of UPF and Water Intake

UPF quartile	Unweighted frequency (n, %)	Weighted frequency (n, %)	Water quartile	Unweighted frequency (n, %)	Weighted frequency (n, %)
1	1,134 (26.4)	36,144,482 (27.9)	1	966 (22.5)	26,910,211 (20.7)
2	1,079 (25.1)	32,235,216 (24.9)	2	1,063 (24.7)	30,063,451 (23.2)
3	1,069 (24.9)	31,326,899 (24.2)	3	1,061 (24.7)	33,138,565 (25.5)
4	1,014 (23.6)	29,974,104 (23.1)	4	1,210 (28.1)	39,731,508 (30.6)
Total	4,296	129,680,701 (100)	Total	4,300	129,843,735 (100)

n: number of subjects; UPF: ultra-processed food.

**Table 3:** Prevalence of PHQ-9–Based Depression by UPF and Water Intake

UPF quartile	Depression (%)	Water quartile	Depression (%)
1	10.7	1	15.4
2	8.7	2	9.0
3	10.7	3	10.2
4	15.4	4	10.9

PHQ-9: Patient Health Questionnaire-9; UPF: ultra-processed food.

**Figure 2:**
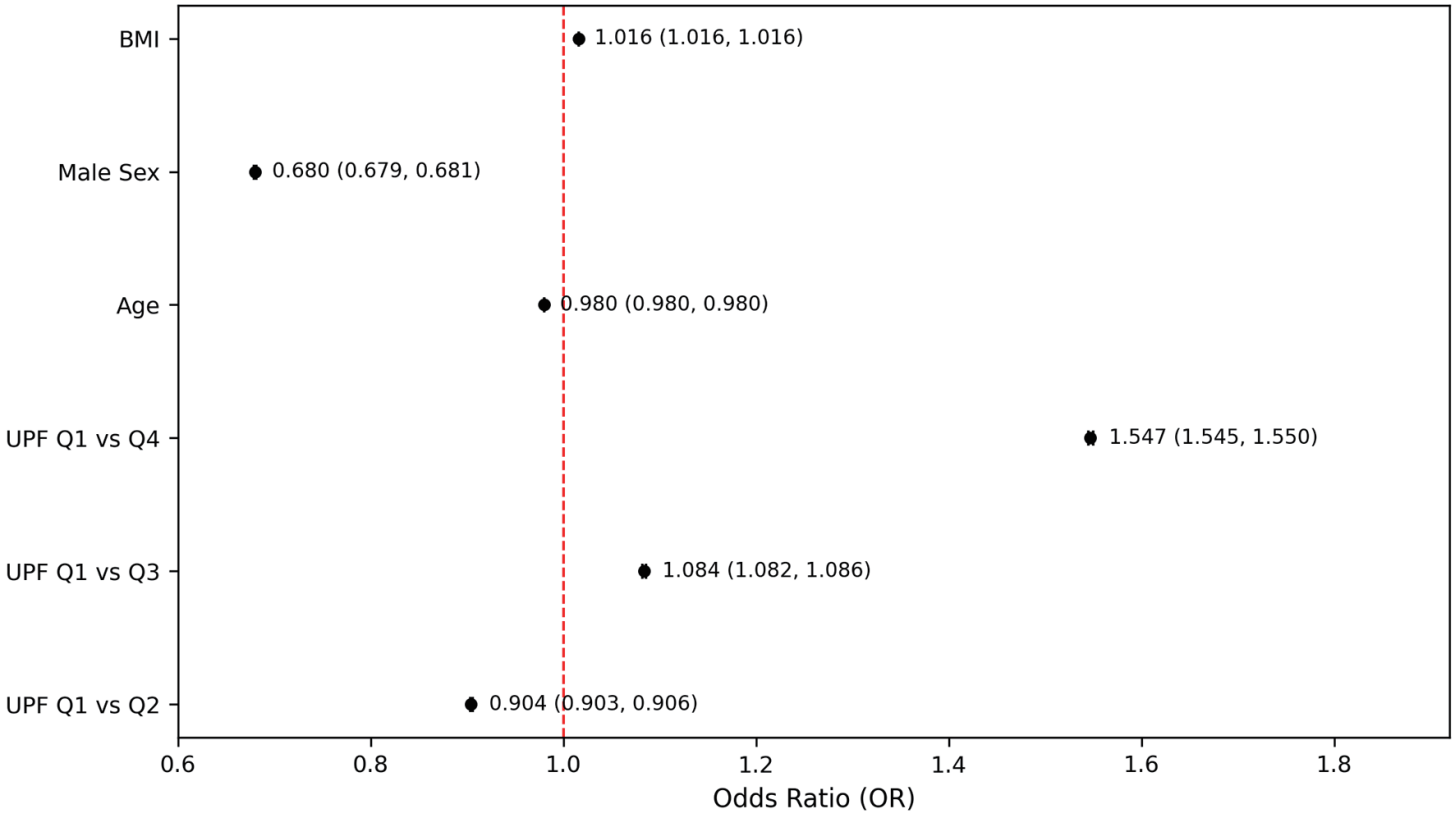
Odds of PHQ-9–based depression by ultra-processed food intake quartiles. Models adjusted for age, sex, and body mass index. PHQ-9: Patient Health Questionnaire-9.

The fully adjusted logistic regression model demonstrated that participants in the highest UPF quartile had about 1.5 times greater odds of reporting moderate-to-severe PHQ-9–based depression compared to those in the lowest UPF quartile (OR = 1.547, 95% CI: 1.545–1.550, P < 0.001) (Fig. 2). Although the odds of PHQ-9–based depression were significantly higher in the highest quartile of UPF intake compared to the lowest, the intermediate quartiles did not exhibit a consistent progression, specifically, the second quartile showed lower odds (OR = 0.904, 95% CI: 0.903–0.906, P < 0.001), while the third quartile showed slightly elevated odds (OR = 1.084, 95% CI: 1.082–1.086, P < 0.001) (Fig. 2). This model also demonstrated that male participants had reduced odds of PHQ-9–based depression compared to females (OR = 0.680, 95% CI: 0.679–0.681, P < 0.001), and for each additional year of age there was a modest decrease in the odds of PHQ-9–based depression (OR = 0.980, 95% CI: 0.980–0.980, P < 0.001) (Fig. 2).

**Figure 3:**
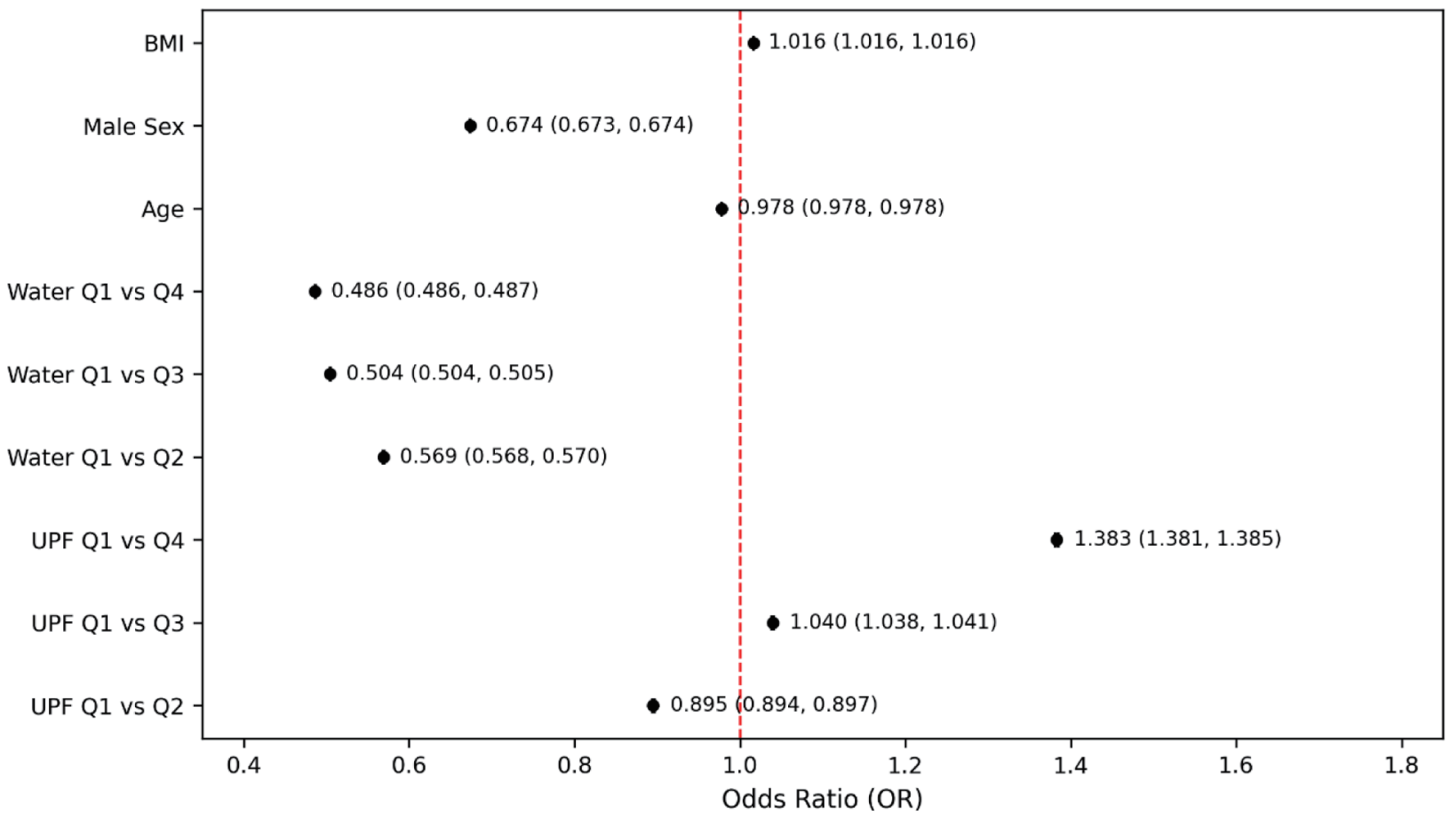
Odds of PHQ-9–based depression by ultra-processed food intake and water intake quartiles. Models adjusted for age, sex, and body mass index. PHQ-9: Patient Health Questionnaire-9.

**Figure 4:**
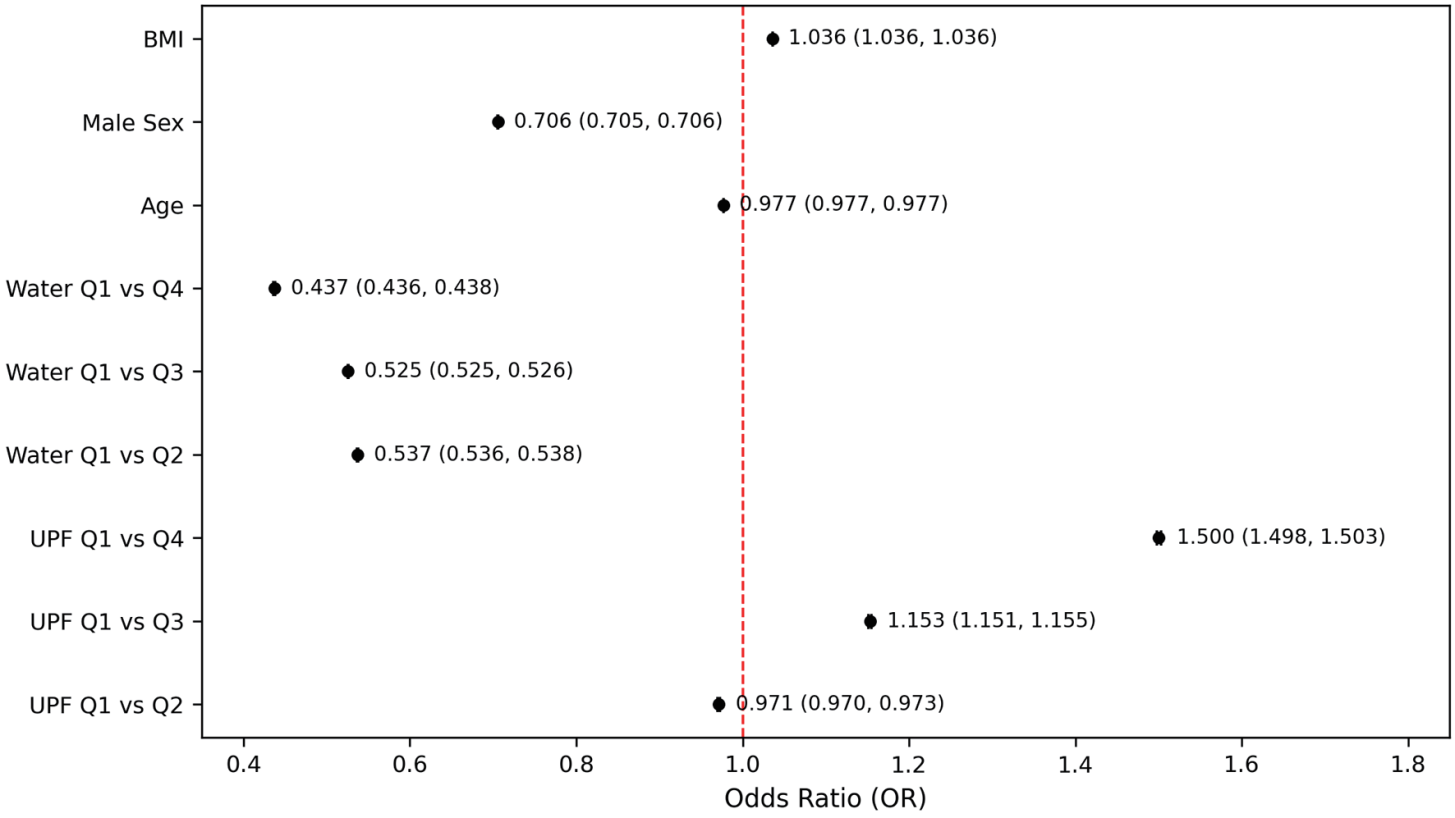
Odds of PHQ-9–based depression by ultra-processed food and water intake quartile after excluding participants with extreme body mass indices. Models adjusted for age, sex, and body mass index. PHQ-9: Patient Health Questionnaire-9.

In the fully adjusted model that included both UPF and water intake quartiles (Fig. 3), participants in the highest quartile of water consumption had significantly lower odds of PHQ-9–based depression compared to those in the lowest quartile (OR = 0.486, 95% CI: 0.486–0.487, P < 0.001). This inverse association remained consistent in stratified and sensitivity analyses. The magnitude of the effect indicated that higher water intake was associated with more than a 50% reduction in the odds of PHQ-9–based depression. The sensitivity analysis (Fig. 4), which excluded participants with extreme BMI values (< 18.5 or > 40 kg/m^2^), further demonstrated the robustness of the association between depression and UPF intake, particularly when compared with the highest and lowest quartiles (OR = 1.500; 95% CI: 1.498–1.503). However, despite this significant finding between quartile extremes, intermediate quartiles showed inconsistent odds, with quartile 2 showing slightly decreased odds (OR = 0.971, 95% CI: 0.970–0.973, P < 0.001) and quartile 3 having modestly increased odds (OR = 1.153, 95% CI: 1.151–1.155, P < 0.001). Conversely, the sensitivity analysis revealed a more consistent and clear inverse relationship between water intake and PHQ-9–based depression. Individuals in higher quartiles of water intake had significantly lower odds of PHQ-9–based depression compared to those in the lowest quartile. Specifically, the ORs were notably decreased and remained consistent across higher water intake quartiles (Q2: OR = 0.537; Q3: OR = 0.525; Q4: OR = 0.437). This pattern demonstrates a steady protective effect, suggesting that greater water intake was associated with a progressively reduced odds of PHQ-9–based depression (Fig. 4).

**Figure 5:**
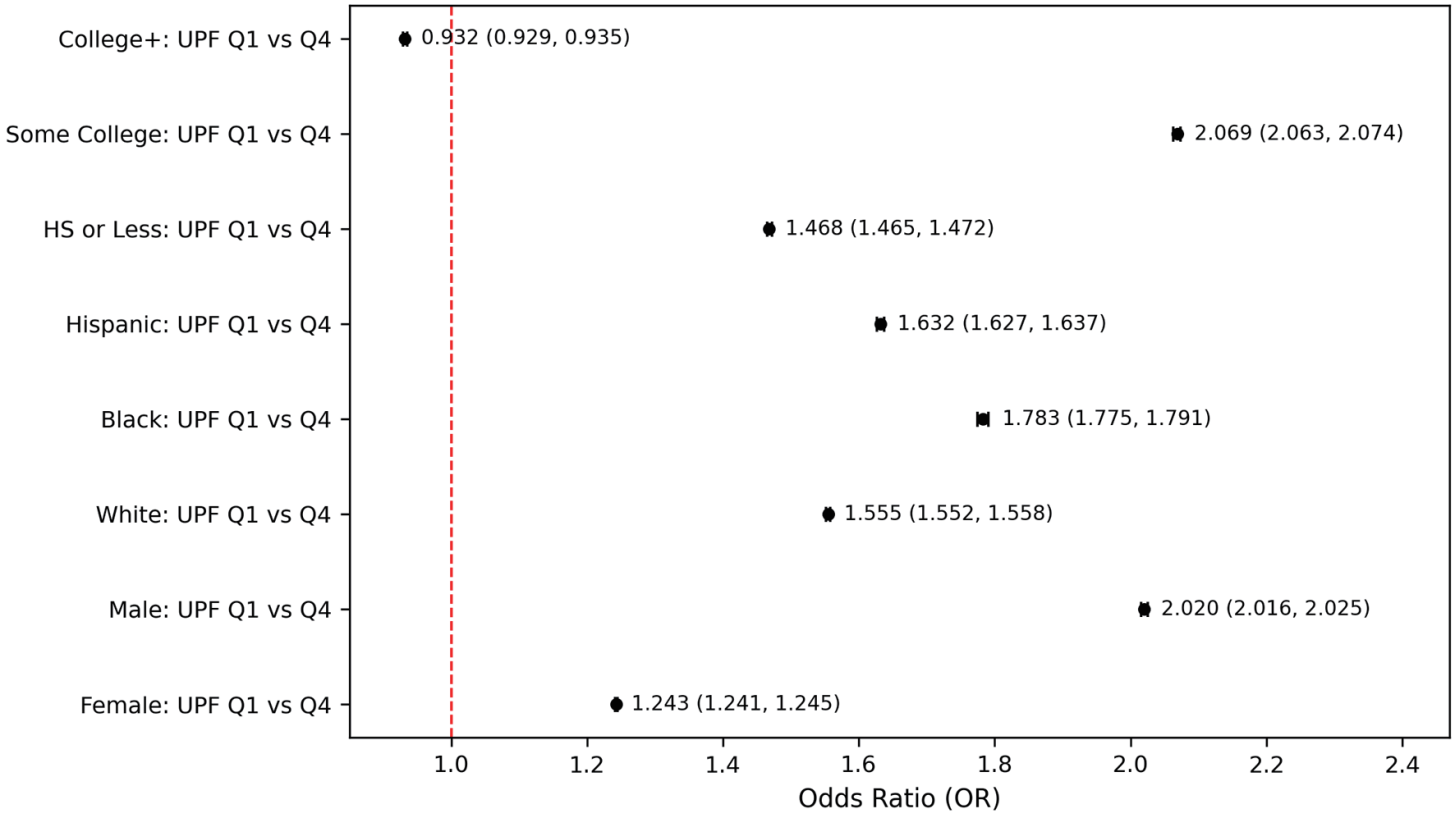
Subgroup-specific odds for PHQ-9–based depression comparing lowest vs. highest UPF intake quartiles. HS: high school level education; PHQ-9: Patient Health Questionnaire-9.

Subgroup-specific analysis revealed differential associations by demographic characteristics. When compared to participants in the lowest quartile and highest quartiles of UPF consumption, there were notable differences across gender, race, and educational attainment (Fig. 5). Among males, the association between UPF intake and PHQ-9–based depression was particularly strong, with those in the highest UPF quartile having an OR of 2.020 (95% CI: 2.016–2.025), compared to an OR of 1.243 (95% CI: 1.241–1.245) in females. Differences were also pronounced across racial and ethnic groups. Race-stratified analyses indicated that Black individuals showed the strongest association between high UPF intake and PHQ-9–based depression (OR = 1.783, 95% CI: 1.775–1.791), while the effect was also moderate in Hispanic individuals (OR = 1.632, 95% CI: 1.627–1.637) and in White individuals (OR = 1.555, 95% CI: 1.552–1.558) (Fig. 5). Stratification by educational attainment revealed a complex pattern. Participants with only some college education had a particularly higher odds of PHQ-9–based depression when consuming the highest levels of UPFs compared to the lowest quartile (OR = 2.069; 95% CI: 2.063-2.074, P < 0.001). Those with a high school education or less also showed a substantial elevation in risk (OR = 1.468, 95% CI: 1.465–1.472, P < 0.001). Individuals with a college education or higher displayed only a slightly reduced odds of PHQ-9–based depression at higher UPF intake levels (OR = 0.932, 95% CI: 0.929–0.935, P < 0.001) (Fig. 5).

**Figure 6:**
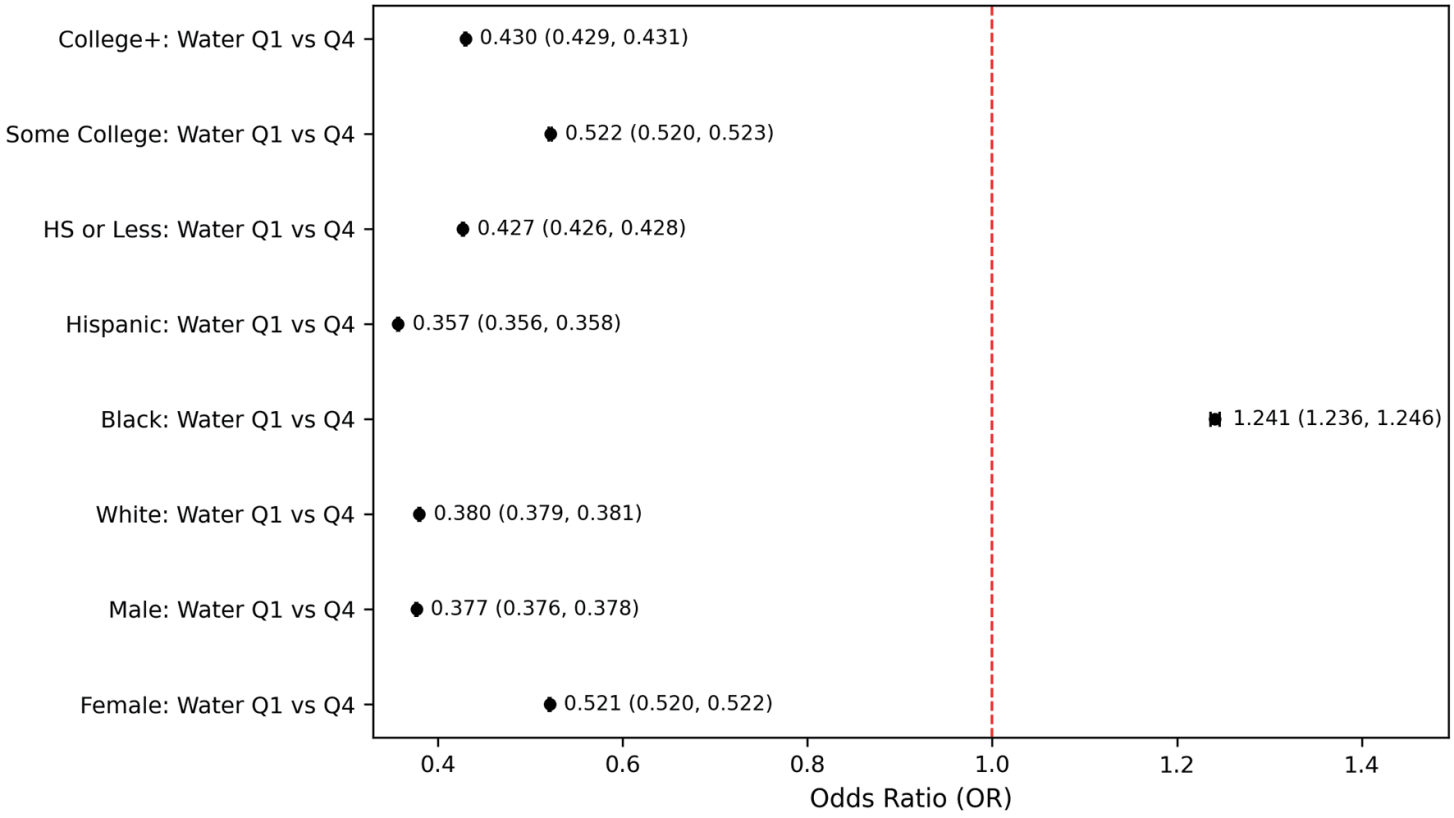
Subgroup-specific Odds for PHQ-9–based depression comparing lowest vs. highest water intake quartiles. HS: high school level education; PHQ-9: Patient Health Questionnaire-9.

Subgroup-specific analysis between water intake and PHQ-9–based depression across demographic characteristics revealed differential associations (Fig. 6). When compared to participants in the lowest versus highest quartiles of water intake, notable variations emerged by gender, race, and educational attainment. Both genders showed significant protective effects of higher water intake, with males demonstrating a notably stronger association, specifically, males in the highest quartile of water intake had substantially reduced odds of PHQ-9–based depression (OR = 0.377, 95% CI: 0.376–0.378, P < 0.001) compared to those in the lowest quartile. Differences across racial and ethnic groups were notable. Hispanic individuals showed the strongest protective effect, with individuals in the highest water intake quartile having substantially reduced odds of PHQ-9–based depression compared to the lowest quartile (OR = 0.357, 95% CI: 0.356–0.358, P < 0.001). Similarly, White individuals exhibited a strong protective effect (OR = 0.380, 95% CI: 0.379–0.381, P < 0.001). In contrast, Black individuals uniquely demonstrated increased odds of PHQ-9–based depression at higher water intake (OR = 1.241, 95% CI: 1.236–1.246, P < 0.001) (Fig. 6). Stratification by education showed consistent protective effects of higher water intake across all educational levels, with some variability in magnitude. Individuals with college education or higher exhibited robust protective effects (OR = 0.430, 95% CI: 0.429–0.431, P < 0.001), closely followed by those with a high school education or less (OR = 0.427, 95% CI: 0.426–0.428, P < 0.001).

In continuous survey-weighted linear regression models (Table 4), higher percentage of calories from added sugars (UPF proxy) was significantly associated with higher log-transformed PHQ-9 scores (β = 0.17; t = 4.51; P < 0.0001), independent of age, sex, and BMI. Total water intake was inversely associated with PHQ-9 scores (β = –0.00; t = –3.23; P = 0.0012). Although the coefficient for water intake was small (rounded to 0.00 in SPSS output), the direction of effect suggests a modest inverse relationship, consistent with approximately a 0.2–0.3 unit lower log PHQ-9 per additional liter of water consumed daily. These results were consistent when participants with extreme BMI values were excluded (Table 4).

**Table 4:** Survey-Weighted Linear Regression of Continuous Predictors of Log-Transformed PHQ-9 Depression Score

Predictor	β estimate	t value	P value
UPF intake	+0.17^a^	4.51	< 0.0001
Water intake (g/day)	0.00^b^	–3.23	0.0012
Age (years)	–0.04	–9.39	< 0.0001
Male (sex)	–0.79	–5.00	< 0.0001
BMI (kg/m^2^)	+0.05	3.98	< 0.0001
UPF × water intake interaction	–0.07	–2.38	0.017

^a^Standardized β = +0.19 in sensitivity model. ^b^Rounded to 0.00 from SPSS output. BMI: body mass index; PHQ-9: Patient Health Questionnaire-9; UPF: ultra-processed food.

**Figure 7:**
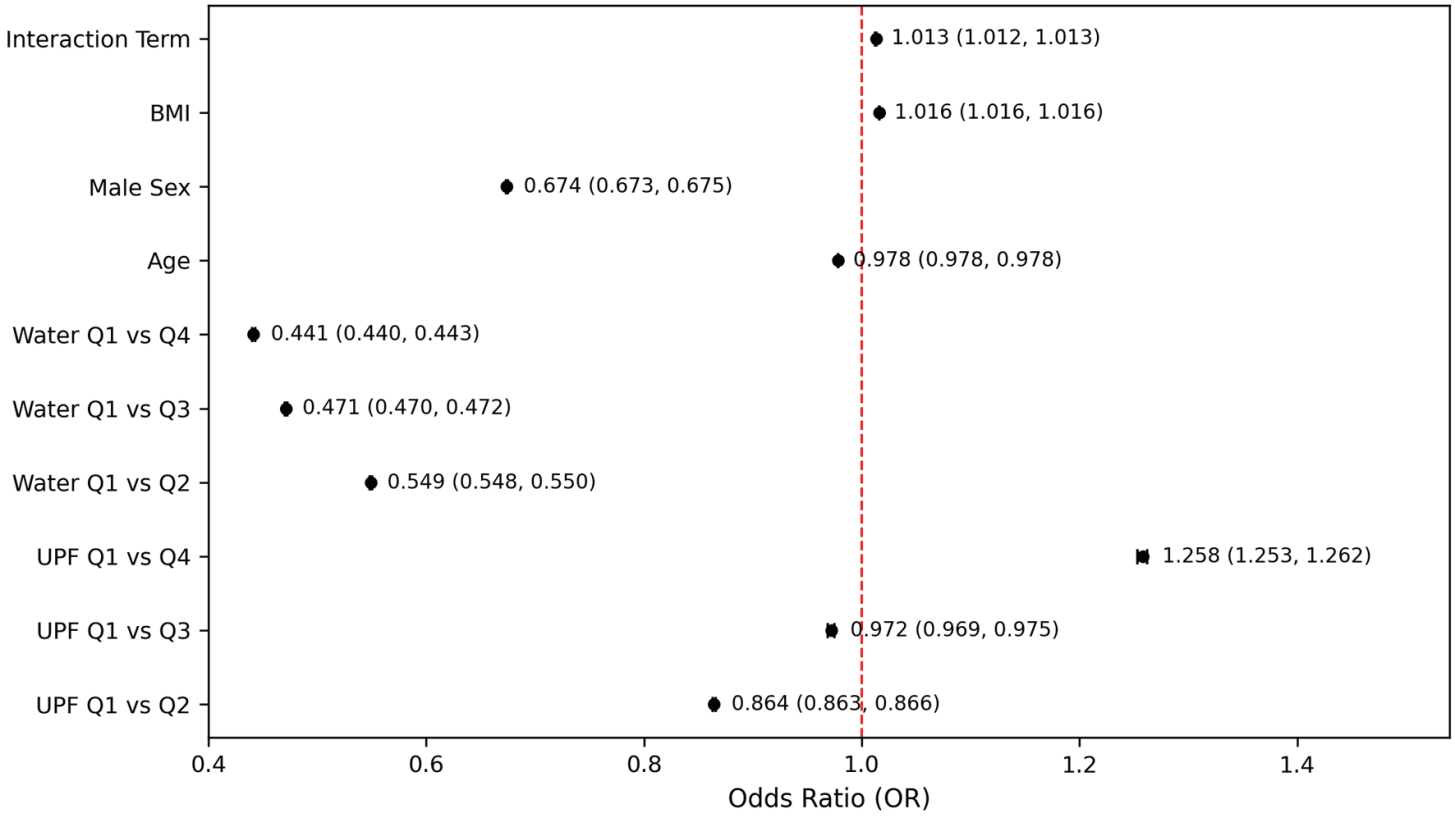
Independent and interactive effects of ultra-processed food and water intake on PHQ-9–based depression. Models adjusted for age, sex, and body mass index. PHQ-9: Patient Health Questionnaire-9.

To explore whether water intake modified the association between UPF consumption and PHQ-9–based depression, an interaction term was included in the regression model (Fig. 7). The interaction between UPF quartile and water intake quartile was statistically significant (OR = 1.013, 95% CI: 1.012–1.013; P < 0.001). However, the effect size was extremely small, suggesting minimal practical or clinical significance. Although statistically significant, the observed interaction odds (OR = 1.013) implied that for each stepwise increase in UPF quartile, the influence of water intake on PHQ-9–based depression risk was only marginally affected which indicated that the protective benefit of higher water intake remained relatively consistent, regardless of the UPF intake level. Specifically, even among those with higher UPF intake, maintaining higher water consumption still provided a clear protective effect against PHQ-9–based depression, indicating that increased hydration was broadly beneficial across different dietary contexts (Fig. 7). In continuous survey-weighted linear regression models (Table 4), when the UPF–water interaction term was included, the moderating effect of water intake remained statistically significant (β = –0.07; t = –2.38; P = 0.017), also indicating that higher water intake attenuated the positive association between UPF consumption and depressive symptom severity.

To assess the robustness of the primary findings to potential confounding by health-related behaviors, we conducted sensitivity analyses that additionally adjusted for smoking status and alcohol consumption. Models were adjusted for age, sex, BMI, race, education level, income-to-poverty ratio, smoking status, and alcohol use. All sensitivity models retained the same survey-weighted logistic regression framework, outcome definition (moderate-to-severe depression defined as PHQ-9 ≥10), exposure definitions (UPF intake quartiles and water intake quartiles), and covariates as the primary analyses, with smoking and alcohol use included as additional covariates. In sensitivity analyses examining UPF intake alone (Table 5), higher UPF consumption remained associated with increased odds of depression after adjustment for smoking and alcohol use. Compared with participants in the lowest UPF quartile (Q1), those in the highest quartile (Q4) had greater odds of depression (OR 1.493; 95% CI: 1.491–1.495). In sensitivity analyses examining water intake alone (Table 6), higher water intake remained inversely associated with depression after adjustment for smoking and alcohol use. Compared with participants in the lowest water intake quartile (Q1), those in the highest quartile (Q4) demonstrated substantially lower odds of depression (OR = 0.526; 95% CI: 0.525–0.527). Similar protective associations were observed for Q1 vs. Q2 (OR = 0.608; 95% CI: 0.607–0.609) and Q1 vs. Q3 (OR = 0.527; 95% CI: 0.526–0.527), indicating a consistent inverse relationship across quartiles.

**Table 5:** Sensitivity Analysis: UPF Quartiles and Depression Adjusted for Smoking and Drinking Alcohol

Variable	P-value	OR (Exp(B))	95% CI
Age	< 0.001	0.978	0.978–0.978
Male (vs. female)	< 0.001	0.618	0.617–0.619
BMI	< 0.001	1.021	1.020–1.021
Drinking alcohol vs. no alcohol	< 0.001	0.640	0.638–0.641
Smoking status			
Never vs. current	< 0.001	2.365	2.361–2.369
Never vs. former	< 0.001	1.257	1.255–1.259
UPF quartile			
Q2 vs. Q1	< 0.001	0.926	0.925–0.928
Q3 vs. Q1	< 0.001	1.115	1.113–1.117
Q4 vs. Q1	< 0.001	1.493	1.491–1.495

BMI: body mass index; CI: confidence interval; OR: odds ratio; UPF: ultra-processed food.

**Table 6:** Sensitivity Analysis: Water Intake Quartiles and Depression Adjusted for Smoking and Drinking Alcohol

Variable	P-value	OR (Exp(B))	95% CI
Age	< 0.001	0.976	0.976–0.976
Male (vs. female)	< 0.001	0.620	0.619–0.620
BMI	< 0.001	1.020	1.020–1.020
Drinking alcohol vs. no alcohol	< 0.001	0.664	0.663–0.665
Smoking status			
Never vs. current	< 0.001	2.161	2.157–2.164
Never vs. former	< 0.001	1.256	1.254–1.258
UPF quartile			
Q2 vs. Q1	< 0.001	0.911	0.910–0.913
Q3 vs. Q1	< 0.001	1.065	1.063–1.067
Q4 vs. Q1	< 0.001	1.346	1.344–1.348
Water quartile			
Q2 vs. Q1	< 0.001	0.608	0.607–0.609
Q3 vs. Q1	< 0.001	0.527	0.526–0.527
Q4 vs. Q1	< 0.001	0.526	0.525–0.527

BMI: body mass index; CI: confidence interval; OR: odds ratio; UPF: ultra-processed food.

In joint sensitivity models including both UPF intake and water intake (Table 7), both exposures retained independent associations with depression after mutual adjustment and adjustment for smoking and drinking alcohol. In this joint model, the association between UPF intake and depression was attenuated but remained directionally consistent, with an OR of 1.043 (95% CI: 1.040–1.047) for Q4 versus Q1. In contrast, the inverse association between water intake and depression remained robust, with ORs of 0.553 (95% CI: 0.552–0.554) for Q2, 0.439 (95% CI: 0.438–0.440) for Q3, and 0.407 (95% CI: 0.406–0.409) for Q4 compared with Q1. The UPF × water interaction term was statistically significant but small in magnitude (OR = 1.034; 95% CI: 1.034–1.035), indicating limited effect modification (Table 7).

**Table 7:** Sensitivity Analysis: Joint UPF and Water Model Adjusted for Smoking and Drinking Alcohol

Variable	P-value	OR (Exp(B))	95% CI
Age	< 0.001	0.976	0.976–0.976
Male (vs. female)	< 0.001	0.619	0.618–0.620
BMI	< 0.001	1.020	1.020–1.020
Drinker (vs. non-drinker)	< 0.001	0.663	0.662–0.664
Smoking status			
Never vs. current	< 0.001	2.174	2.171–2.177
Never vs. former	< 0.001	1.257	1.255–1.258
UPF quartile			
Q2 vs. Q1	< 0.001	0.828	0.826–0.829
Q3 vs. Q1	< 0.001	0.888	0.886–0.891
Q4 vs. Q1	< 0.001	1.043	1.040–1.047
Water quartile			
Q2 vs. Q1	< 0.001	0.553	0.552–0.554
Q3 vs. Q1	< 0.001	0.439	0.438–0.440
Q4 vs. Q1	< 0.001	0.407	0.406–0.409
UPF × water interaction	< 0.001	1.034	1.034–1.035

BMI: body mass index; CI: confidence interval; OR: odds ratio; UPF: ultra-processed food.

## Discussion

This cross-sectional analysis of NHANES 2021–2023 data provides robust evidence that UPF intake and plain water consumption are independently associated with the risk of moderate-to-severe depression in the US adults. Individuals with the highest UPF intake had significantly greater odds of depression, whereas those with the highest water intake showed substantially reduced odds compared to their respective lowest quartiles. These associations persisted after adjusting for critical confounders including age, sex, smoking and alcohol use, and BMI, and were consistent across sensitivity and subgroup analyses. In sensitivity analyses additionally adjusting for smoking status and alcohol consumption, the associations between UPF intake, water intake, and depressive symptoms remained directionally consistent with the primary models. Although effect estimates were modestly attenuated after inclusion of these lifestyle behaviors, higher UPF intake continued to be associated with increased odds of depression. Participants in the highest UPF quartile had approximately 50% higher odds of depression compared with those in the lowest quartile, even after adjustment. Moreover, higher water intake remained inversely associated with depression after adjustment. Participants in the highest water intake quartile had nearly 60% lower odds of depression compared with those in the lowest quartile. These findings suggest that smoking and alcohol use account for some shared behavioral risk but do not fully explain the observed relationships between diet composition, hydration, and depression. The persistence of these associations in joint models further supports the interpretation that UPF intake and water intake represent distinct, independent contributors to depression risk rather than proxies for other unhealthy behaviors. The modest magnitude of the UPF × water interaction indicates limited effect modification, with primary associations largely preserved across intake levels.

A study used a similar methodology and found comparable results regarding consumption of UPFs and depression, but it only included analysis years between 2007 and 2012 [21]. The COVID-19 pandemic marks the most significant discontinuity in data collection over the decade; while one study found a clear increase in cases of major depression by 27% globally post-pandemic [22], the effects of the pandemic on the overall consumption of UPFs seem more heterogenous. While many population studies, particularly in Brazil, reported increased UPF intake during the pandemic, some adolescent and international cohorts, namely in the US, Greece, Sweden, and Korea, showed a modest or even sustained decline [23–27]. Moreover, from 2012 to 2021, NHANES remained methodologically rigorous but adapted to pandemic-related interruptions, introduced modified weighting for analytic validity, and expanded its health surveillance scope [28–30].

The findings from this NHANES analysis contribute to a growing body of literature implicating UPFs in adverse mental health outcomes. Mechanistic hypotheses point toward increased systemic inflammation, microbiome dysregulation, and glycemic instability as potential mediators of this relationship [8–11]. Conversely, the observed protective effect of water intake is relatively novel in population-based data and aligns with existing literature suggesting that hydration supports optimal neuronal and cognitive function. While the correlation between UPF and water intake was statistically significant (r = 0.162), its weakness underscores the independence of these behaviors, each potentially modifiable through distinct public health strategies.

We found that the subgroup analyses exposed striking demographic disparities in vulnerability. In contrast to the current literature on gender disparities in depression, male participants exhibited some of the strongest associations between high UPF intake and PHQ-9–based depression. Existing literature consistently reports that women have a higher prevalence of major depressive disorder than men, a disparity that begins in adolescence and persists across the lifespan. These gender differences are thought to arise from a complex interplay of biological factors, hormonal fluctuations, psychological vulnerabilities, and social stressors, though findings on symptom expression and treatment response remain mixed and context-dependent [31–34]. However, our analysis showed that with regard to UPF intake, in men, higher UPF intake exerted stronger association with PHQ-9–based depression. We also found that while high UPF intake was consistently associated with increased PHQ-9–based depression risk across all race categories analyzed, the magnitude of this association varied in the form that Black and Hispanic individuals exhibited comparatively greater vulnerability, with stronger associations between high UPF consumption and PHQ-9–based depression relative to other groups. Racial disparities in depression treatment utilization among Black and Hispanic individuals are influenced by a range of interconnected factors [35–38]. These include cultural beliefs and preferences that shape attitudes toward mental health care, persistent stigma surrounding mental illness, especially within minority communities, provider-level implicit and explicit biases that affect clinical decision-making, and broader systemic barriers embedded in health care structures, communities, and policies that continue to reinforce inequities in access and treatment options [35–38]. Education-stratified results suggested that individuals with only a high school diploma or some college experience were more susceptible to diet-related mood disturbances than their college-educated peers. The existing literature seems to support this inverse relationship between depression and educational attainment, with the relationship likely being bidirectional [39–42]. These findings highlight the intersection of nutrition, sociodemographic context, and mental health, providing opportunities for more targeted, culturally informed interventions.

Despite the strengths of this study, namely a nationally representative large sample and rigorous adjustment for confounding variables, several limitations merit discussion. First, the cross-sectional design precludes causal inference. Although the associations are statistically robust and biologically plausible, reverse causality cannot be excluded; individuals with depression may be more likely to consume UPFs or neglect hydration. Second, UPF intake was proxied by the percentage of calories from added sugars, which, while a reasonable approximation based on previous literature [12, 13], does not fully capture the complexity of food processing classification as outlined in the Nova system. While the percentage of calories from added sugars was used as a proxy for UPF intake, we acknowledge that more granular food-level data might exist that could allow for direct application of the Nova classification system to NHANES dietary records. However, this process involves time-intensive food code mapping and the use of external classification algorithms that are not yet fully integrated with the 2021–2023 dietary cycles. Given the strong correlation between UPF intake and added sugar consumption in the US diets, with prior studies showing that UPFs account for nearly 90% of added sugar intake, we believe this proxy offers a pragmatic and interpretable measure of UPF exposure at the population level [12, 13]. Nevertheless, we recognize that this approach may lead to some exposure misclassification and recommend future analyses applying full Nova-based classification as a sensitivity check or validation step. Similarly, water intake was based on self-reported dietary recall, which is subject to measurement error and recall bias.

Another limitation lies in residual confounding. Although the models adjusted for age, sex, BMI, race, ethnicity, and educational status, other influential factors such as, physical activity, medication use, and comorbid medical or psychiatric conditions, were not included. Omission of these variables might have confounded the observed associations. Finally, the clinical significance of the UPF × water intake interaction is limited despite reaching statistical significance; the marginal effect size (OR = 1.013) suggests minimal practical impact.

Future research studies are needed to prioritize longitudinal designs for better assessment of temporality and causality. Prospective cohort studies or randomized dietary interventions that concurrently manipulate UPF consumption and water intake would provide more definitive evidence. Moreover, incorporating biomarker-based hydration measures and more nuanced dietary intake assessments (e.g., Nova classification, food diaries) could improve exposure measurement. Stratified analyses by income, geographic region, or dietary patterns (e.g., Western vs. Mediterranean) could further elucidate context-specific vulnerabilities.

### Conclusion

Increased UPF intake is associated with higher risk of depression in the US adults while increased water intake confers a protective effect. These findings underscore the need for dietary strategies that simultaneously reduce UPF intake and promote hydration, with tailored interventions for the high-risk groups.

## Data Availability

The authors declare that data supporting the findings of this study are available within the article.
